# Low-dose interferon-*γ-*producing human neuroblastoma cells show reduced proliferation and delayed tumorigenicity

**DOI:** 10.1038/sj.bjc.6601842

**Published:** 2004-05-04

**Authors:** I Airoldi, R Meazza, M Croce, E Di Carlo, T Piazza, C Cocco, T D'Antuono, V Pistoia, S Ferrini, M V Corrias

**Affiliations:** 1 Laboratory of Oncology, Gaslini Institute, Largo Gaslini 5, 16148 Genoa, Italy; 2 Laboratory of Clinical and Experimental Immunology, Gaslini Institute, Largo Gaslini 5, 16148 Genoa, Italy; 3 Laboratory of Immunopharmacology, Istituto Nazionale per la Ricerca sul Cancro, Largo Benzi 12, 16132 Genoa, Italy; 4 Department of Oncology and Neurosciences, G. D'Annunzio University, 66100 Chieti, Italy

**Keywords:** neuroblastoma, IFN-*γ*, transfection, cancer vaccine, tumour gene therapy

## Abstract

Interferon-*γ* (IFN-*γ*) directs T helper-1 cell differentiation and mediates antitumour effects in preclinical models. However, high-dose IFN-*γ* is toxic *in vivo*, and IFN-*γ*-transfected neuroblastoma (NB) cells secreting high amounts of the cytokine may be lost due to cell apoptosis or differentiation. Two human NB cell lines (ACN and SK-N-BE2(c)) differing as to genetic and phenotypic features were transfected with the human IFN-*γ* gene and selected on the grounds of the low concentrations of IFN-*γ* produced. In both IFN-*γ*-transfected cell lines, autocrine and paracrine activation of IFN-*γ*-mediated pathways occurred, leading to markedly reduced proliferation rate, to increased expression of surface HLA and CD40 molecules and of functional TNF binding sites. ACN/IFN-*γ* cells showed a significantly delayed tumorigenicity in nude mice as compared to parental cells. ACN/IFN-*γ* tumours were smaller, with extensive necrotic area as a result of a damaged and defective microvascular network. In addition, a significant reduction in the proliferation index was observed. This is the first demonstration that IFN-*γ* inhibits *in vivo* proliferation of NB cell by acting on the tumour cell itself. This effect adds to the immunoregulatory and antiangiogenic activities operated by IFN-*γ* in syngeneic tumour-bearing hosts.

Neuroblastoma (NB), the most frequent extracranial solid tumour of childhood, arises from the neural crest, and the prognosis for patients presenting with disseminated disease at diagnosis is poor despite multimodal aggressive therapy (Brodeur *et al*, 1993).

Immunisation of cancer patients with cytokine-engineered tumour cells has been investigated at preclinical and clinical levels (Forni and Boggio, 1999; Pardoll, 2000; Parmiani *et al*, 2000; Wadhwa *et al*, 2002).

Several preclinical studies, performed in syngeneic mice with murine NB cell lines engineered to express different cytokines (Watanabe *et al*, 1989; Katsanis *et al*, 1994; Bausero *et al*, 1996), HLA antigens (Hock *et al*, 1995,^1996^; Heuer *et al*, 1996;) or costimulatory molecules (Katsanis *et al*, 1995; Heuer *et al*, 1996; Katsanis *et al*, 1996; Grossmann *et al*, 1997), have shown the therapeutic efficacy of this approach. In a xenogeneic model, we have previously shown that IL-2-transfected human NB cells inhibited the growth of parental tumour cells coinjected in nude mice (Corrias *et al*, 1998).

So far, three different phase I trials have been performed in NB patients unresponsive to conventional treatments, by employing either autologous or allogeneic NB tumour cells engineered to produce IL-2, alone or in combination with lymphotactin (Brenner *et al*, 2000; Rousseau *et al*, 2003; Bowman *et al*, 2003). In these trials, some of the vaccinated patients showed increase in leucocyte count, antibody levels and in NK activity.

Among the cytokines so far investigated, interferon (IFN)-*γ* appears to be of particular interest due to its ability to upregulate surface expression of HLA class I molecules in human NB cells (Lampson *et al*, 1983; Ponzoni *et al*, 1993; Corrias *et al*, 2001) and to activate cytotoxic T-lymphocyte-mediated antitumour responses in a murine model (Watanabe *et al*, 1989).

However, studies in humans have shown that high-dose recombinant IFN-*γ* administration is associated with serious side effects. Local low-dose release of cytokines, obtainable by gene transfer, may overcome systemic toxicity without impairing immune recognition of malignant cells by the immune system. In small cell lung carcinoma, a tumour that displays a downregulation of HLA class I antigen and shares neuroectodermal origin with NB, IFN-*γ* transfection have been demonstrated to enhance HLA class I surface expression and restore presentation of a MAGE-3 peptide to CTL (Traversari *et al*, 1997).

This approach appears feasible also for human NB cells since NB tumours express CTL-defined TAA, such as tyrosine hydroxylase ((Lode *et al*, 2000), MAGE-1 and 3 (Rimoldi *et al*, 1993; Corrias *et al*, 1996), NY-ESO (Soling *et al*, 1999; Rodolfo *et al*, 2003) and ALK (Lamant *et al*, 2000), and tumour-specific cytolytic T lymphocytes have been isolated from NB-bearing patients (Rosenthal *et al*, 1994; Bowman *et al*, 1998; Sarkar *et al*, 2001; Rousseau *et al*, 2003).

In addition to serving as an immunostimolatory molecule and exerting antiangiogenic effects through IP-10 production (Sgadari *et al*, 1996), IFN-*γ* can sensitise tumour cells to apoptosis (Fulda and Debatin, 2002). Furthermore, it can induce expression of functional surface CD40 molecules on NB cells (Airoldi *et al*, 2003), making them susceptible to CD40L-mediated apoptosis.

In this study, we have established and characterised *in vitro* two low-dose IFN-*γ*-producing NB cell lines, which differ in their genetic and phenotypic features. Furthermore, we have tested tumorigenicity of *ACN/IFN-γ* cell lines in a xenogeneic transplant model in nude mice and have addressed the mechanisms for delayed tumorigenicity.

## MATERIALS AND METHODS

### Vectors and cell lines transfection

The human IFN-*γ* cDNA was amplified by RT–PCR starting from 1 *μ*g of total RNA extracted from PHA-activated peripheral blood lymphocytes. The sequences of PCR primers were: *forward* TGACAGGCTTAATTCTCTCGGAAACG, *reverse* TAGACTTAGGATCCAATATTGCAGGCAGGACAACC; a *Bam*HI site is underlined. The cDNA was cloned in the *Xba*I blunted-*Bam*HI sites of plasmid RSV.5 neo (kindly provided by Dr EO Long, NIH, Bethesda, MD, USA). Both ACN and SK-N-BE2(c) cell lines were transfected with 5 *μ*g of the RSV.5IFN-*γ* (later refered as *ACN/IFN-γ* and *SK/IFN-γ*) or RSV.5 (later refered as *ACN/neo and SK/neo*) using cationic liposomes (DOTAP, Roche, Milano, Italy), according to instructions provided by the manufacturer. Stable transfectants were selected by growing the transfected cells in a medium containing 500 *μ*g ml^−1^ of G-418 (Calbiochem, La Jolla, CA, USA).

### RNA extraction, RT–PCR analysis and RNAse protection assay (RPA)

Total RNA was extracted using the RNeasy extraction kit (Qiagen, Hilden, Germany). In all, 1 *μ*g of total RNA, resuspended in sterile water, was retrotranscribed with oligo d(T) by means of a commercial kit according to the procedure suggested by the manufacturer (Clontech, Palo Alto, CA, USA). At the end of the synthesis, the cDNA was diluted to 100 *μ*l and then 5 *μ*l was separately amplified with primers specific for IFN-*γ*, IP-10, IRF-1, NY-ESO, MAGE-1 and 3, TH, MYC-N, ALK and for the housekeeping gene G3PDH. The primers sequences, positions and accession number of the different sequences, size of the amplification products and annealing temperature are given in Table 1

**Table 1:** Sequence of primers used in RT–PCR analysis and size of the amplification products

**Gene**	**Accession no.**	**Forward**	**Position**	**Reverse**	**Position**	**Temp**	**Bp**
IFN-*γ*	X13274	5′ATTGAATTCACGATGAAATATACAAGTTA	106–125	5′-ATTCTCGAGCAGGCAGGACAACCATTACT	605–624	60°C	518
IP-10	X02530	5′-GGAACCTCCAGTCTCAGCACC	46–66	5′-GGATGATGAACATTAACCTTCC	536–559	58°C	513
IRF-1	NM_002198	5′-GCTCCACTCTGCCTGATGACC	658–678	5′-GAGGAATAAGAGGGGCCCAGG	1183–1203	59°C	545
TH	NM_000360	5′TGTCAGAGCTGGACAAGTGT	528–547	5′-GATATTGTCTTCCCGGTAGC	826–807	60°C	299
MAGE1	AY148486	5′-CGGCCGAAGGAACCTGACCCAG	1–22	5′-GCTGGAACCCTCACTGGGTTGCC	399–421	60°C	420
MAGE3	HSU03735	5′-TGGAGGACCAGAGGCCCCC	2276–2294	5′-GGACGATTATCAGGAGGCCTGC	3059–3080	60°C	804
NY-ESO	NM_001327	5′GCCGCCTGCTTGAGTTCTAC	342–361	5′-CTCCTCCAGCGACAAACAAT	718–737	60°C	395
MYC-N	NM_005378	5′TGTCGGTTGCAGTGTTGGAGG	22–42	5′-TGTCGGTTGCAGTGTTGGAGG	247–267	60°C	245
ALK	XM_055726	5′-GCTGAGCAAGCTCCGCACCTCGAC	4144–4167	5′-CCCGCCATGAGCTCCAGCAGGATG	4486–4509	60°C	365
GAPDH	NM_002046	5′-ACATCGCTCAGAACACCTATGG	16–37	5′-GGGTCTACATGGCAACTGTGAG	1191–1212	60°C	1196

. All the amplifications were performed for 35 cycles, after 10 min at 94°C to activate AmpliTaq gold polymerase (Perkin-Elmer Apllied Biosystem, Norwalk, CT, USA). Amplified products were analysed in a 2% agarose gel run in TBE buffer and stained with ethidium bromide. Specificity of the amplified products was determined by direct sequencing performed with the use of the Dye Terminator Cycle Sequencing Kit (ABI PRISM; Perkin-Elmer Applied Biosystem). Sequences were resolved and analysed on the ABI373A Sequence Apparatus (Perkin-Elmer Applied Biosystem).

RNAse protection assay was performed with 5 *μ*g lane^−1^ total RNA and the Pharmingen probe hCR-3 kit (Pharmingen, San Diego, CA, USA), according to manufacturer's protocol. Products were resolved on 6% denaturing polyacrylamide gels and the protected fragments were visualised and quantitated using a PhosphorImager 445SI (Molecular Dynamics, Sunnyvale, CA, USA).

### Flow cytometry and cell cycle analysis

Surface expression of HLA molecules was analysed by indirect immunofluorescence and cytofluorimetric analysis using the W6.32 mAb (anti HLA I, kindly provided by Dr S Ferrone, Roswell Park Cancer Center, Buffalo NY, USA) and the D1.12 mAb (anti HLA II, kindly provided by Dr R Accolla, University of Insubria, Italy). An FITC-conjugated goat anti mouse IgG2a was used as second-step reagent. Surface expression of CD40 was analysed by means of PE-conjugate anti CD40 mAb from Diaclone SA, Besançon, France. Controls for anti HLA I and HLA II were purified murine IgG2a (Southern Biotechnologies Associates, Birmingham, AL, USA) of irrelevant specificity. Control for anti-CD40 was PE-conjugated, isotype-matched mAb of irrelevant specificity. Intracellular staining for anti Ki-67 was performed by incubating cells in PBS containing 0.1% saponin and anti Ki-67 antibody (clone MIB-1, Dako, Glostrup, Denmark) or the corresponding control isotype (purified murine IgG1 of irrelevant specificity from Southern Biotechnologies Associates). An FITC-conjugated goat anti mouse IgG1 was used as second-step reagent. Samples were analysed with the FACScan (BD Biosciences-Mountain View, CA, USA). Cells were scored using a FACScan analyzer (Becton-Dickinson, San Jose, CA, USA) and data were processed using CellQuest software (Becton-Dickinson). The threshold line was based on the maximum staining obtained with irrelevant isotype-matched mAb, used at the same concentration as test mAb. Negative cells were defined such that less than 1% of cells stained positive with control mAbs. Cells labeled with test antibody that were brighter than those stained with isotypic control antibody were defined as positive.

Cell cycle analysis was performed by incubating 10^5^ cells in PBS containing 40 *μ*g ml^−1^ propidium iodide, 0.1% Triton X-100 and 400 *μ*g ml^−1^ RNase A for 10 min at 37°C. Cells were analysed by flow cytometry using CellQuest software, after 10 additional minutes at room temperature.

### Cytokine production

Supernatants of semiconfluent, G418-resistant, IFN-*γ*-transfected NB cells were collected after 48 h and analysed for human IFN-*γ* production by a commercially available ELISA kit (R&D System, Minneapolis, MN, USA). Cells were counted and results are referred to 48 h production from 10^6^ cells.

### TNF-*α* receptor binding assay

Cells were seeded in triplicate at a density of 10^6^ well^−1^ in 2 ml medium in six-well plates. After 24 h, cells were washed twice and then incubated for 2 h at 4°C in cold medium containing 80–120 pM ^125^I-labeled hrTNF-*α* (Sorin Pharmaceuticals, Verona, Italy) in the presence or absence of increasing concentrations of unlabelled hrTNF-*α* (a generous gift of Boehringer Mannheim, Mannheim, Germany). Afterwards, cells were washed twice with ice-cold medium, detached with ice-cold PBS and washed twice by centrifugation. The pellet-associated ^125^I was counted in a *γ*-counter (LKB, Uppsala, Sweden) with an estimated efficiency of 80%. Cell number was determined by counting cells in a hemocytometer. To evaluate the binding affinity and number of site per cell, the ligand displacement curves were subjected to Scatchard analysis by means of the Ligand program by considering TNF-*α* as a trimer, as previously described (Montaldo *et al*, 1994).

### Coculture of parental and IFN-*γ*-transfected cells

Parental cells (5 × 10^5^) were seeded on the bottom plate and 5 × 10^5^ of either the empty vector- or the IFN-*γ*-transfected cells were seeded on the upper plate of a transwell (Costar, Cambridge, MA, USA). The two chambers were separated by a policarbonate filter with pore size of 0.4 *μ*m. Afterdays of coculture, the upper well was discarded and the parental cells on the bottom plate were detached and analysed by flow cytometry or by RT–PCR analysis.

### Nude mice studies

Pathogen-free female athymic (nu/nu) mice, 6–8 weeks old, were obtained from Harlan Italy (San Pietro al Natisone, Italy). Animal experiments, performed according to the National Regulation on Animal Research Resources, were approved by the Review Board of the Istituto Nazionale per la Ricerca sul Cancro. Mice were housed under sterile conditions and received autoclaved food and water. Animals (seven for each group) were injected subcutaneously with 2 × 10^7^ parental ACN or transfected *ACN/neo* or *ACN/IFN-γ* cells. Tumour size, measured twice a week with a caliper, was expressed as a multiple of the wider and smaller diameters. Statistical analysis was performed by the Mann–Withney test.

### Morphological analysis

*ACN/neo* and *ACN/IFN-γ* tumours were removed at day 14 post injection (p.i.), fixed in 10% neutral-buffered formalin, embedded in paraffin, sectioned at 4 *μ*m, and stained with haematoxylin–eosin for histological evaluation. For immunohistochemistry, formalin-fixed, paraffin-embedded sections were incubated with anti-human Ki-67 (clone MIB-1, Dako) or anti laminin (Ab No. 078P, BioGenex, San Ramon, CA, USA) Abs. The rates of proliferating (immunoreactivity for Ki-67) cells were obtained by counting the number of positive cellsper number of total cells in the viable neoplastic tissue excluding areas of tissue necrosis under a microscope at × 400 in a 0.180 mm^2^ field. Differences in the number of Ki-67-positive cells were evaluated by Student's *t*-test.

## RESULTS

### Stable IFN-*γ* transfection of NB cell lines

Several NB cell lines were transfected with the recombinant RSVneo/human IFN-*γ* gene or the empty vector. All the transfectants producing IFN-*γ* concentrations in culture supernatants higher than 25 pg ml^−1^ 48 h 10^6^ cells^−1^were excluded from the study. Two stable, low-dose IFN-*γ*-producing transfected cell lines, ACN and SK-N-BE2(c), were established. ACN cells lack MYC-N amplification and 1p3.6 deletion (Thiele, 1999), but express MAGE-1 and -3 (Corrias *et al*, 1996), as well as NY-ESO and ALK (this paper, see below). In addition, ACN cells are tumorigenic in nude mice (Corrias *et al*, 1998). On the contrary, SK-N-BE2(c) cells are MYC-N amplified and 1p deleted (Thiele, 1999), express MYC-N and TH (this paper, see below) and are not tumorigenic in nude mice.

**Figure 1:**
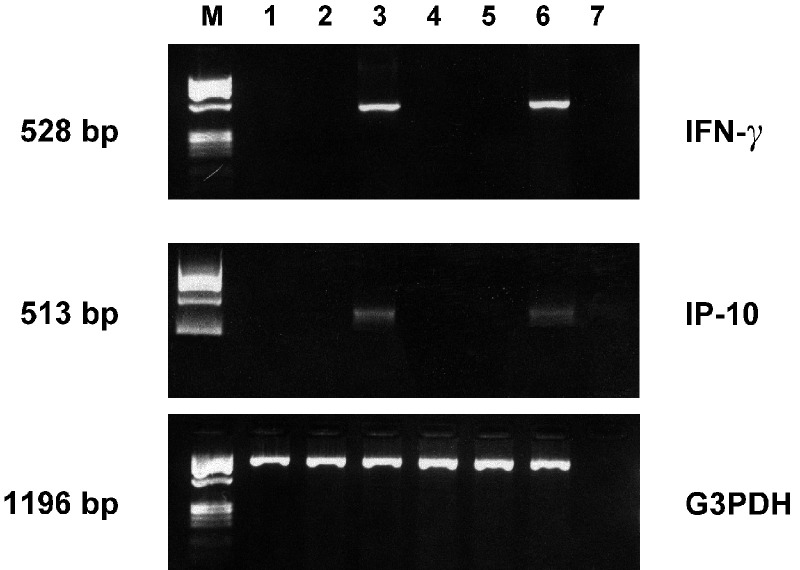
IFN-*γ* and IP-10 gene expression in parental and IFN-*γ* -transfected NB cell lines. RT–PCR analysis performed on parental, empty vector- and IFN-transfected SK-N-BE2(c) (lanes 1–3) and ACN (lanes 4–6) cells, and water as negative control (lane 7). Size of the expected fragments is indicated on the left. G3PDH amplification products are shown in the lower panel. M=Φ*χ*174 *Hae*/digest molecular weigh marker.

Both stable IFN-*γ* transfectants expressed human IFN-*γ*-specific transcripts when tested by RT–PCR (Figure 1

). IFN-*γ* production in culture supernatants from IFN-*γ*-transfected ACN and SK-N-BE2(c) cells was 19.2 and 18.5 pg ml^−1^ 48 h^−1^ 10^6^ cells^−1^, respectively, as assessed by ELISA.

### *In vitro* characterisation of IFN-*γ* transfectants

IFN-*γ*-transfected cells from both the cell lines displayed a more differentiated phenotype, that is presence of neurites, than their parental counterparts (data not shown), as observed following treatment with hr IFN-*γ* (Ponzoni *et al*, 1992).

**Figure 2:**
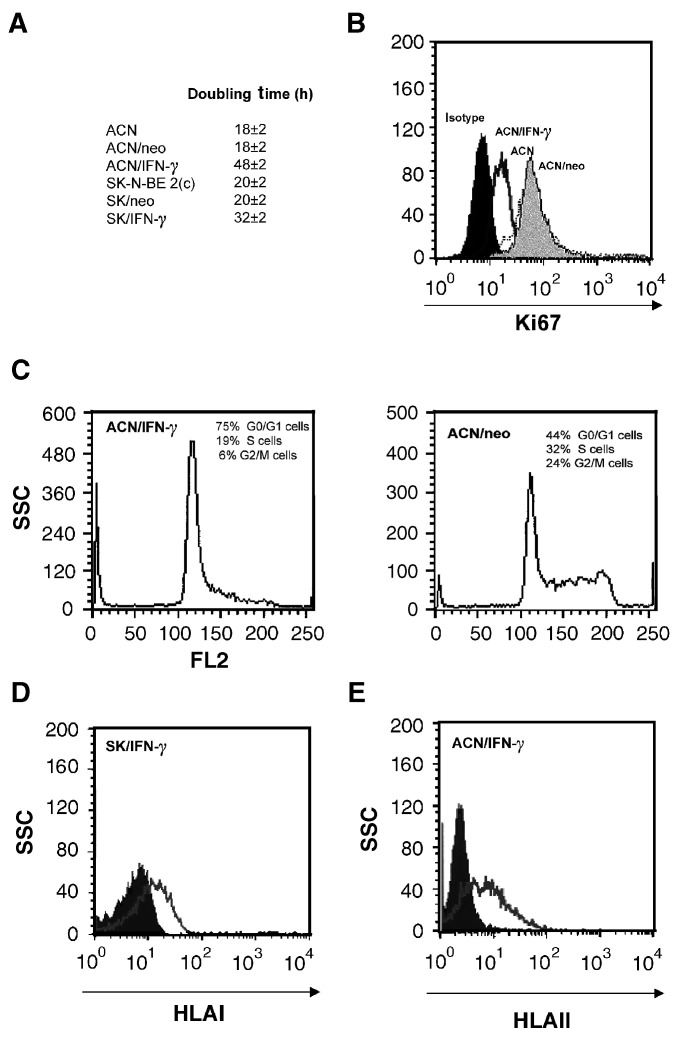
*In vitro* effects of IFN-*γ* transfection on NB cells. (**A**) Doubling time of IFN-*γ-*transfected, empty vector-transfected and parental NB cells, assessed by counting cells every day for a week (**B**) Proliferation index of ACN, *ACN/IFN-γ* and *ACN/neo*, assessed by Ki-67 staining. (**C**) Cell cycle analysis of *ACN/IFN-γ* (left panel) and *ACN/neo* (right panel) cells. (**D**) HLA class I surface expression of *SK/IFN-γ* cells (dark profile: *SK/neo*; open profile: *SK/IFN-γ*). (**E**) HLA class II surface expression of *ACN/IFN-γ* cells (dark profile: *ACN/neo*; open profile: *ACN/IFN-γ*), as assessed by flow cytometry.

IFN-*γ* transfection activated IFN-regulated pathways in both NB cell lines, as indicated by *de novo* expression of the IFN-inducible IP-10 gene transcript (Figure 1). Moreover, both *ACN/IFN-γ* and *SK/IFN-γ* transfectants displayed a dramatic increase in their doubling time, as evaluated by cell count (Figure 2A

). In order to understand the mechanism(s) underlying cell growth inhibition, we analysed the effect of released IFN-*γ* on proliferation rate and apoptosis. IFN-*γ* transfection significantly (*P*<0.005) reduced the proliferation rate, assessed by Ki-67 staining, of *ACN/IFN-γ* cells, as compared to parental and vector-transfected cells (Figure 2B). Indeed, cell cycle analysis showed that *ACN/IFN-γ* cells were mostly arrested in G0/G1 phase (Figure 2C), as compared to vector-transfected *ACN/neo* cells. On the contrary, IFN-*γ* transfection did not significantly modify the amount of apoptotic cells in culture (data not shown). Similar results were obtained with the SK-N-BE2(c) transfectants (data not shown).

IFN-*γ* transfection induced, in both cell lines, remarkable changes in surface expression of HLA molecules. In the SK-N-BE2(c) cell line, which constitutively expresses very low amounts of HLA class I and no HLA class II molecules (Corrias *et al*, 2001), IFN-*γ* transfection produced a clear-cut increase in HLA class I antigen (Figure 2D) and no changes in HLA class II expression (not shown) with respect to *SK/neo* cells. In the ACN cell line, which constitutively expresses HLA class I and low amounts of HLA class II molecules (Corrias *et al*, 2001), increased expression of HLA class II antigens was detected (Figure 2E) with respect to the *ACN/neo* cells.

Consistent changes also occurred in the surface expression of TNF-*α* binding sites, as assessed by Scatchard analysis using radiolabelled hrTNF-*α* (Ponzoni *et al*, 1992; Montaldo *et al*, 1994). As shown in Table 2

**Table 2:** Growth rate, TNF receptor surface expression and effect of TNF-*α* treatment on growth rate of parental, vector- and IFN-transfected NB cells

		**Proliferation**		
	**TNF-*α* binding sites per cell^a^**	**Medium^b^**	**+TNF-*α*^b^**	**Mean percentage of inhibition**
ACN	310±15	4732±216	3332±287	24.8
ACN/neo	306±16	4545±244	3572±196	22.3
*ACN/IFN-γ*	942±36	3837±322	2243±312	41.5
SK-N-BE 2(c)	129±11	4955±269	1944±103	58.3
SK/neo	132±12	4643±268	2187±259	57.4
*SK/IFN-γ*	467±26	4051±266	1598±327	69.5

a see Materials and Methods for details on Scatchard analysis.

b 1 h [^3^H]thymidine incorporation measured in 5 × 10^4^ cells cultured for 2 days in medium or in medium containing hrTNF-*α* at 100 IU ml^−1^.

All the results are the mean±s.d. of three independent experiments, each carried out in duplicate.

, IFN-*γ*-transfected cells expressed three times more binding sites than parental and empty vector-transfected cells. Furthermore, when the IFN-*γ* transfectants were treated with hrTNF-*α*, their growth rate was further reduced (Table 2). Inhibition of cell proliferation was higher than that observed in the parental or empty vector-transfected cells (Table 2), although it was more evident for *ACN/IFN-γ* cells than for *SK/IFN-γ* cells. These findings confirmed the occurrence of a synergistic effect between IFN-*γ* and TNF-*α* at inducing differentiation of NB cells (Ponzoni *et al*, 1992; Montaldo *et al*, 1994).

### Effect of IFN- *γ* released by IFN-*γ* transfectants on cocultured parental cells

To test whether the low amounts of IFN-*γ* released by IFN-*γ* transfectants was sufficient to upregulate, by a paracrine mechanism, IFN-*γ*-inducible gene expression in the parental cells, coculture in a transwell system was performed.

**Figure 3:**
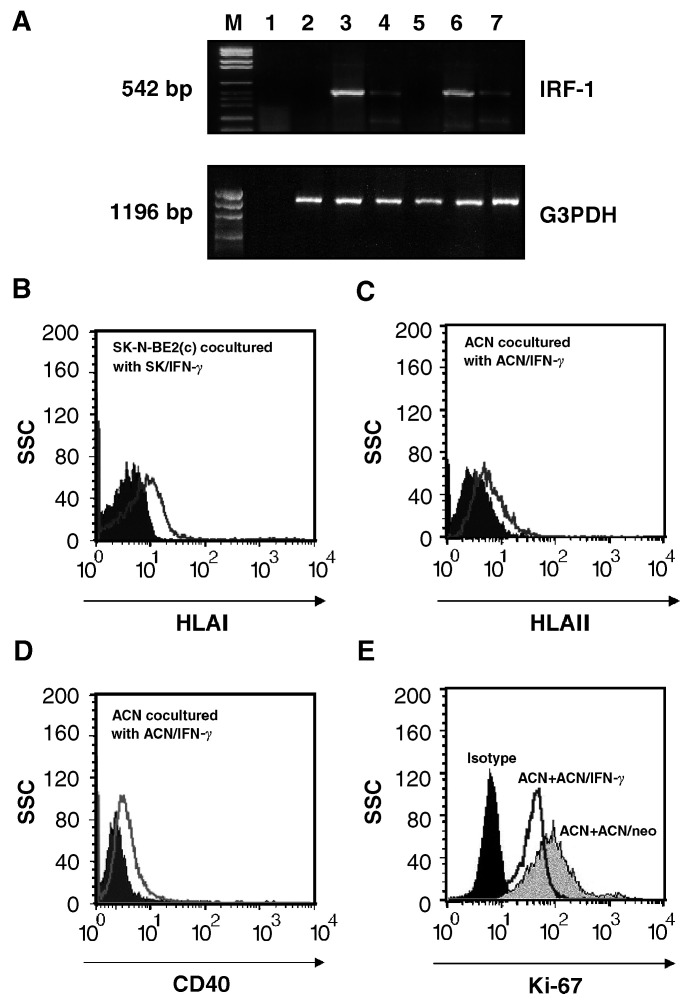
Effects of IFN-*γ* transfection on cocultured parental NB cells. (**A**) RT–PCR analysis of IRF-1 gene expression. M=molecular weigh markers. Negative control (lane 1), ACN cells (lane 2), *ACN/IFN-γ* cells (lane 3), ACN cells cocultured with *ACN/IFN-γ* cells (lane 4), SK-N-BE2(c) cells (lane 5), *SK/IFN-γ* cells (lane 6), SK-N-BE2(c) cocultured with *SK/IFN-γ* cells (lane 7). G3PDH gene expression is shown below. Size of the expected fragment is indicated on the left side. (**B**) HLA class I surface expression of parental SK-N-BE2(c) cells cocultured with *SK/IFN-γ* cells for 72 h (dark profile: irrelevant murine IgG2a; open profile anti HLA I W6.32 mAb). (**C**) HLA class II surface expression of parental ACN cells cocultured with *ACN/IFN-γ* cells for 72 h (dark profile: irrelevant murine IgG2a; open profile: anti HLA II D1.12 mAb). (**D**) CD40 surface expression of parental ACN cells cocultured with *ACN/IFN-γ* cells for 72 h (dark profile: PE-conjugated mAb of irrelevant specificity; open profile: PE-conjugated anti CD40 mAb). (**E**) Ki-67 staining of ACN cells cocultured with *ACN/IFN-*γ (open profile) or *ACN/neo* (grey profile), as assessed by flow cytometry. Dark profile: ACN cells stained with an isotype-matched irrelevant mAb.

Both parental cell lines showed induction of IRF-1 gene expression, as assessed by RT–PCR analysis, upon coculture for 3 days with the corresponding IFN-*γ* transfectants (Figure 3A

). IRF-1 is the transcription factor necessary and sufficient to enhance expression of all the IFN-*γ*-inducible genes (Loh *et al*, 1992). Following coculture with paired IFN-*γ* transfectants, parental SK-N-BE2(c) cells expressed HLA class I molecules (Figure 3B) and parental ACN cells HLA class II molecules (Figure 3C). No modification in HLA surface expression was observed when the parental ACN and SK-N-BE2(c) cells were cocultured with the empty vector-transfected cells (not shown).

We have recently demonstrated that treatment of several NB cell lines with hrIFN-*γ* induced surface expression of CD40 (Airoldi *et al*, 2003). *ACN/IFN-γ* also showed enhanced CD40 gene and surface expression (Airoldi *et al*, 2003). *ACN/IFN-γ* cells cocultured with parental ACN cells induced CD40 surface expression on these latter cells (Figure 3D), further confirming that a paracrine effect was produced by the low amount of IFN-*γ* released by the NB transfectants. Similar results were obtained when ACN cells were cocultured with *SK/IFN-γ* cells and, conversely, the latter cells were cocultured with *ACN/IFN-γ* cells (data not shown).

Finally, IFN-*γ* released by *ACN/IFN*-*γ* transfectants significantly reduced (*P*<0.005) the proliferation rate of cocultured parental ACN cells, as assessed by Ki-67 staining (Figure 3E). No change in the percentage of apoptotic cells was observed under this conditions (data not shown). Similar results were obtained in parental SK-N-BE2(c) cells cocultured with *SK/IFN*-*γ* (data not shown).

### Expression of TAA genes in IFN-*γ*-transfected NB cells

**Figure 4:**
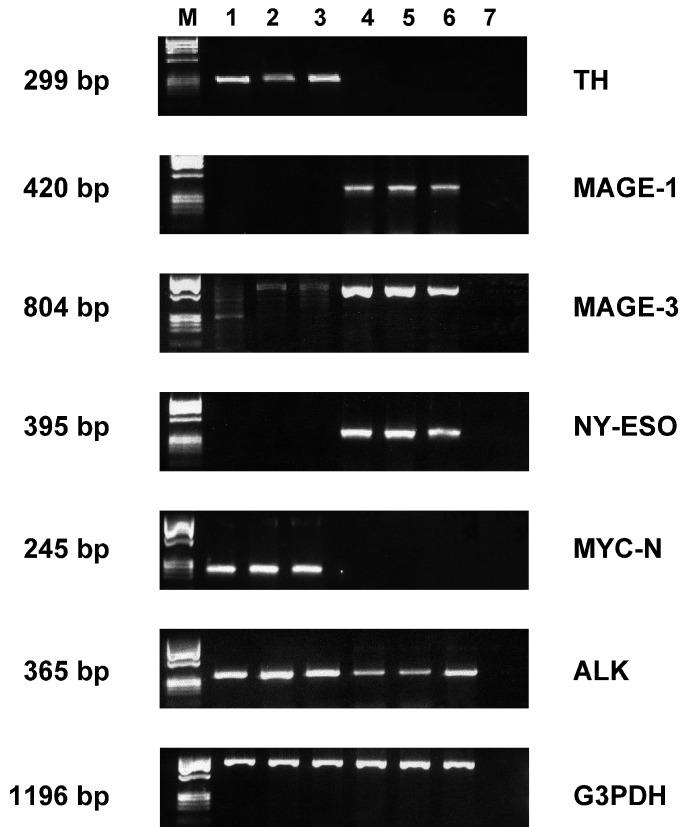
TAA gene expression in parental and transfected NB cell lines. RT–PCR analysis performed on parental, empty vector- and IFN-transfected SK-N-BE2(c) (lanes 1–3) and ACN (lanes 4–6) cells and water as negative control (lane 7). Size of the expected fragments is indicated on the left. G3PDH amplification products are shown in the lower panel. M=Φ*χ*140 Hae/digest molecular weigh marker.

Next, we investigated the effects of IFN-*γ* transfection on the expression of different TAA genes, as assessed by RT–PCR. As shown in Figure 4

, *SK/IFN-γ* cells contained the transcripts of MYC-N, ALK and TH genes as parental SK and SK*/neo* cells, while *ACN/IFN-γ* cells displayed expression of ALK, NY-ESO, MAGE-1 and -3 mRNAs as parental ACN and *ACN/neo* cells (Figure 4).

### Expression of cytokine receptor genes in IFN-*γ*-transfected NB cells

**Figure 5:**
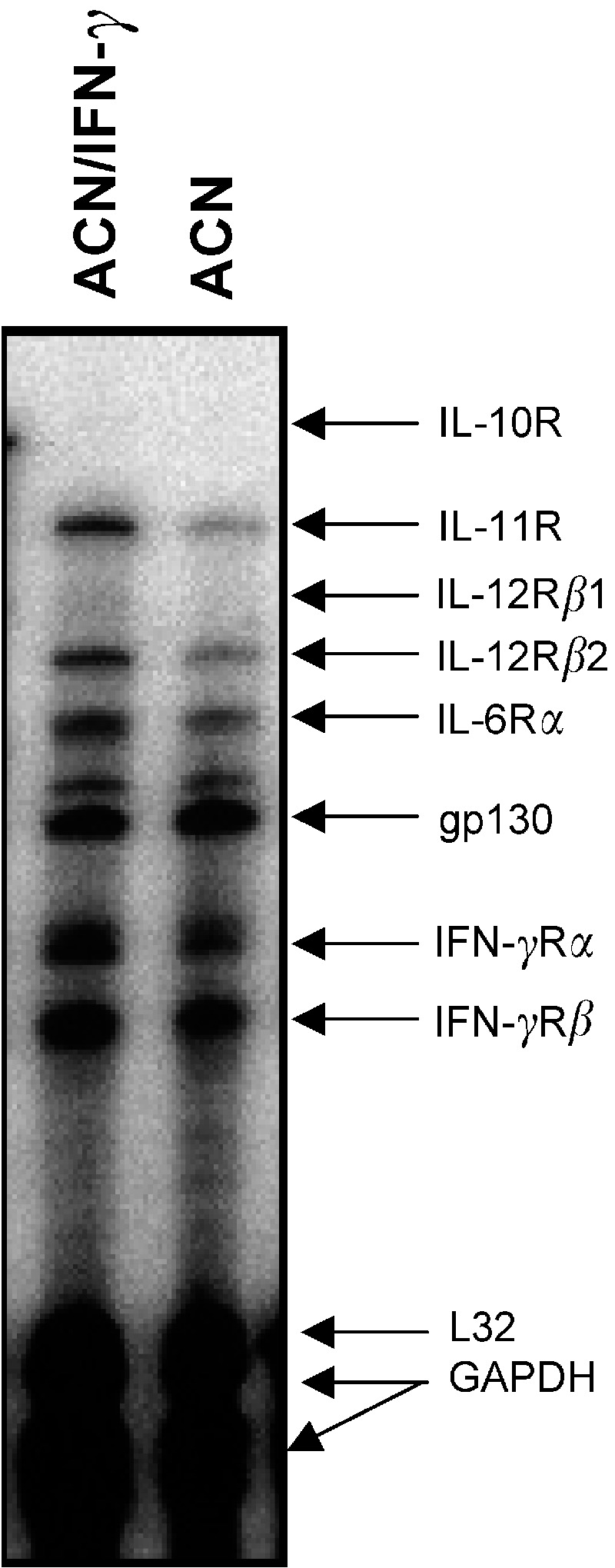
Cytokine receptor gene expression in parental and IFN*γ*-transfected ACN cells as assessed *by* RPA. RNase protection assay was performed with 5 *μ*g lane^−1^ total RNA and the Pharmingen probe hCR-3 kit.

We next tested by RNAse protection assay whether IFN-*γ* transfection modified gene expression of different cytokine receptors, including IFN-*γ* itself. As shown in Figure 5

, *ACN/IFN-γ* cells showed enhanced mRNA expression of IFN-*γ* receptor (R) *α*, IL-6R*α*, IL-11R and IL-12R*β*2. Notably, ACN and SK-N-BE2(c) cells did not express IL-12R*β*1, irrespective of IFN-*γ* gene transfection (Figure 5). In addition, expression of two proinflammatory cytokines, IL-8 and TNF-*α*, known to be expressed by NB cell lines (Nitta *et al*, 1994; Yang *et al*, 1994), and of two anti-inflammatory cytokines, IL-10 and TGF-*β*1, known to inhibit antitumour responses (Hsieh *et al*, 2000) was not modified by IFN-*γ* gene transfection (data not shown).

### Tumorigenicity of ACN/IFN-*γ* in nude mice and histopathological features of tumours

**Figure 6:**
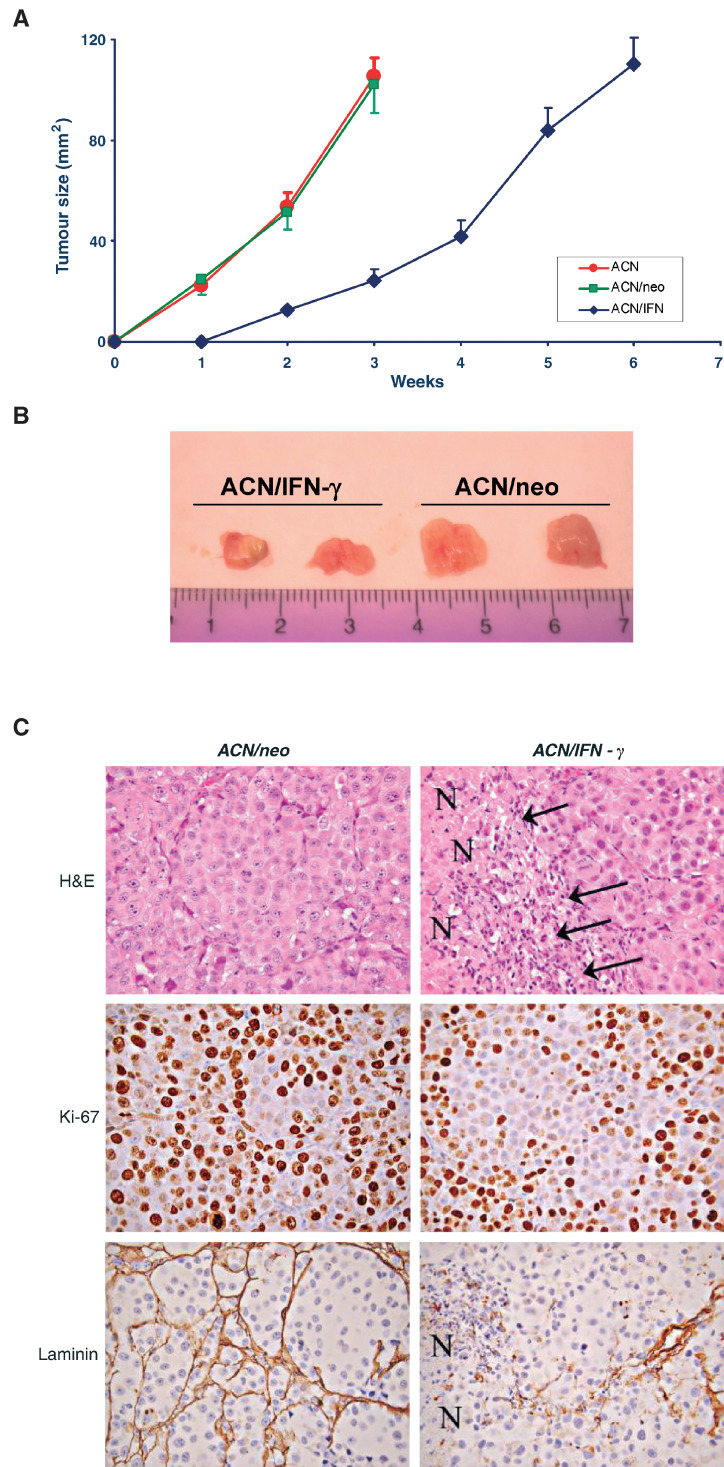
Growth rate, histological and immunohistochemical features of parental, ACN/neo and ACN/IFN-*γ* cells in nude mice. (**A**) Growth rate *in vivo*. 2 × 10^7^ parental (○-○), vector-transfected (□-□) or IFN-transfected ACN (◊-◊) cells were inoculated subcutaneously in the left flank of nude mice. Tumour growth was measured twice a week with a caliper. Results are the mean of two independent experiments, each one performed with five animals per group. (**B**) Photograph of *ACN/neo and ACN/IFN-γ* tumours taken 14 days post injection (p.i.). (**C**) Histological and immunohistochemical analysis of *ACN/neo* and *ACN/IFN-γ* tumours developed 14 days p.i. H&E: *ACN/neo* and *ACN/IFN-γ* tumour sections stained with haematoxylin–eosin. Small nests of round to oval cells with abundant amphophilic cytoplasm and nucleolated nuclei are shown. *ACN/IFN-γ* tumours showed extensive areas of necrosis (N) frequently infiltrated by reactive cells (arrows). Ki-67: Tumour sections stained with anti Ki-67 mAb. Laminin: Tumour sections stained with anti-laminin mAb. Magnification is × 400.

Species specificity of IFN-*γ* and IP-10 does not allow one to test elicitation of antitumour responses in immunodeficient mice bearing human IFN-*γ*-transfected cells. Therefore, we tested whether IFN-*γ* transfection affected the tumorigenicity of ACN cell line by acting through autocrine and paracrine mechanisms at the level of the tumour cell themselves. Parental, *ACN/neo* and *ACN/IFN*-*γ* cells were injected subcutaneously in nude mice and tumour growth was measured. The *in vivo* growth of *ACN/IFN*-*γ* cells was significantly (*P*<0.005) slower than that of parental or vector-transfected ACN cells *in vivo* (Figure 6A

). To address whether this was a consequence of reduced proliferation rate, histopatological analyses were performed on tumours removed at days 14 post injection, when macroscopic differences in the volume of the *ACN/IFN-γ* and *ACN/neo* tumours were observed (Figure 6B).

The tumour masses developed 14 days after s.c. injection of *ACN/neo* cells into nu/nu mice were formed by small nests of closely packed round or oval cells with abundant amphophilic cytoplasm and vesciculated nuclei containing one or more conspicuous nucleoli (Figure 6C, H&E). The pattern of growth was vaguely nodular as a result of the presence of delicate, incomplete fibrous septa. Small necrotic foci were a rather constant feature. However, mitotic figures were also frequent (Figure 6C, H&E).

Mice injected with *ACN/IFN-γ* cells developed tumours, which were smaller in size (Figure 6B) and showed extensive necrotic areas (Figure 6C, H&E, N) often infiltrated with granulocytes and macrophages phagocyting cell debris (Figure 6C, H&E arrows). The proliferation index, assessed by Ki-67 immunoreactivity in the viable neoplastic tissue excluding areas of tissue necrosis, was significantly lower (*P*<0.005) than in *ACN/neo* tumours (66.5±4.0 *vs* 80.2±5.4%) (Figure 6C, Ki-67).

In addition, staining for laminin, a marker of the basement membrane, revealed that the vascular architecture was mostly intact in *ACN/neo* tumours, while aspects of focal basement membrane destruction and alterations in the microvascular architecture were frequent in the inner portion of *ACN/IFN-γ* tumour (Figure 6C, Laminin).

## DISCUSSION

In this study, two human NB cell lines differing in their genetic and phenotypic features were transfected with the human IFN-*γ* gene. These transfectants, which produced low amounts of the cytokine in culture supernatants, displayed dramatic reduction in their proliferation rate, increased expression of functional TNF-*α* binding sites, HLA and CD40 surface molecules, similarly to that observed *in vitro* following treatment of NB cell lines with hrIFN-*γ* (Ponzoni *et al*, 1992; Montaldo *et al*, 1994; Airoldi *et al*, 2003).

More interestingly, all these changes occurred, by a paracrine mechanism, in parental cells cocultured with the IFN-*γ* transfectants. In addition, IFN-*γ*-transfected ACN cells showed a significantly reduced tumorigenicity, as compared to parental and empty vector-transfected cells. This result was obtained in a model, that is, human tumour cell xenograft in nude mice, which precludes any effect of the transfected cytokine on the mouse immune system, due to the species specificity of human IFN-*γ*. Thus, the slow proliferation rate of NB cells observed *in vivo* was attributable to autocrine and paracrine effects of the transfected cytokine on the tumour cells themselves.

Since human IFN-*γ* is species specific, the antiangiogenic effect we observed likely resulted from anti-angiogenic mediators produced by the tumour cells. The defective intratumoral vascular network may lead to extensive necrosis followed by reactive influx of phagocytosing cells. These results indicated that IFN-*γ* transfection of human NB cells may achieve not only activation of immune antitumour responses, as already demonstrated by others, but also affect NB tumour growth *per se*, by inhibiting NB proliferation and exploiting an antiangiogenic effect.

Murine and human IFN-*γ* gene-engineered NB cells have already been produced (Watanabe *et al*, 1989; Coze *et al*, 1995; Katsanis *et al*, 1995; Bausero *et al*, 1996). Murine IFN-*γ*-transfected NB cells were shown to induce specific CTL effectors in tumour-bearing syngeneic mice (Watanabe *et al*, 1989). So far, human IFN-*γ*-transduced NB cells have been characterised *in vitro* for phenotypic features (Coze *et al*, 1995; Ucar *et al*, 1995) and ability to stimulate alloreactive T-cell responses in mixed lymphocyte/tumour cell cultures (Coze *et al*, 1995; Coll *et al*, 1997).

The strategy of selecting low producing IFN-*γ*-transfected NB cells for this study was followed to prevent loss of transfectants, as already described by others (Coze *et al*, 1995; Ucar *et al*, 1995), due to the potent induction of NB tumour cell differentiation (Ponzoni *et al*, 1992; Ponzoni *et al*, 1993; Montaldo *et al*, 1994) and sensitisation to apoptosis (Fulda *et al*, 2001; Banelli *et al*, 2002; Fulda and Debatin, 2002) caused by high level secretion of the cytokine. The IFN-*γ*-transfected NB cell lines described here have been maintained in culture for prolonged period of time without substantial changes in their characteristics.

Minimal amounts of IFN-*γ* were indeed sufficient to induce remarkable changes in surface expression of several molecules, such as HLA, TNF-R and CD40, in cocultured parental NB cells throughout paracrine mechanism. In this respect, we have recently shown that incubation of CD40-positive NB cells with soluble or insoluble CD40L leads to tumour cell apoptosis (Airoldi *et al*, 2003). These observations may be relevant in the perspective of the design of a vaccination protocol for   patients using allogeneic cell lines transfected with the human IFN-*γ* gene. Thus, low-dose IFN-*γ* secreted by the latter cells locally injected at the tumour resection site (where a few tumour cells may escape surgical intervention) may promote presentation of peptide-derived tumour-associated antigen to T lymphocytes leading to specific anti NB responses. In addition, CD40L-induced apoptosis of tumour cells may contribute to their elimination, provided that CD40L-positive activated T lymphocytes are recruited to the tumour site.

Furthermore, low secretion of IFN-*γ* may help limit *in vivo* side effects elicited by high doses of the cytokine, such as systemic toxicity and altered T-lymphocyte polarisation and/or proliferation, as recently reported by Refaeli *et al* (2002).

Another issue here investigated is the expression of different cytokine receptors in IFN-*γ*-transfected cells. Notably, IL-11R and IL-6R transcripts were clearly upregulated in transfected cells; both IL-6 and IL-11 belong to the gp-130-associated family of neuroactive cytokines. Furthermore, upregulation of the transcripts for both chains of the IFN-*γ*R was observed in transfected cells, in accordance with previous studies. These results indicate that introduction of exogenous IFN-*γ* gene in NB cells may modify their sensitivity to other cytokines, possibly present in the tumour microenvironment.

In conclusion, IFN-*γ*-transfected allogeneic NB cell lines may be envisaged as a delivery system of the cytokine to residual tumour cells in a clinical setting. Low-dose IFN-*γ* secretion would limit its systemic toxicity while retaining most immunomodulatory and antiangiogenic activities.
